# Structural aspects of leg-to-gonopod metamorphosis in male helminthomorph millipedes (Diplopoda)

**DOI:** 10.1186/1742-9994-8-19

**Published:** 2011-08-22

**Authors:** Leandro Drago, Giuseppe Fusco, Elena Garollo, Alessandro Minelli

**Affiliations:** 1 Department of Biology, University of Padova, I 35131 Padova, Italy

**Keywords:** post-embryonic development, segment differentiation, sexual appendages

## Abstract

**Background:**

In the adult males of helminthomorph millipedes, one or two pairs of legs in the anterior part of the trunk are strongly modified into sexual appendages (gonopods) used for sperm transfer during the copula. Gonopods differentiate in an advanced phase of post-embryonic development, in most cases as replacement for the walking legs of the seventh trunk ring, as these first regress to tiny primordia, to eventually develop into gonopods at a subsequent stadium. These extremely localized but dramatic changes have been described as a non-systemic metamorphosis. In the present study we describe morphological and anatomical changes of trunk ring VII associated with non-systemic metamorphosis in four helminthomorph species.

**Results:**

As documented here for the first time by means of traditional histology methods and new techniques based on confocal laser scanning microscopy, the external modifications caused by non-systemic metamorphosis are associated to a huge rearrangement of internal anatomy, mostly due to the development of gonopod apodemes and extrinsic muscles.

**Conclusions:**

Internal changes in the seventh trunk ring, locally leading to the dorsal displacement of the ventral nerve cord and the digestive tract, are modulated in a taxon-specific manner, and are very conspicuous in the blaniulids *Nopoiulus kochii *and *Blaniulus guttulatus*, with likely major functional consequences.

## Background

**Figure 1:**
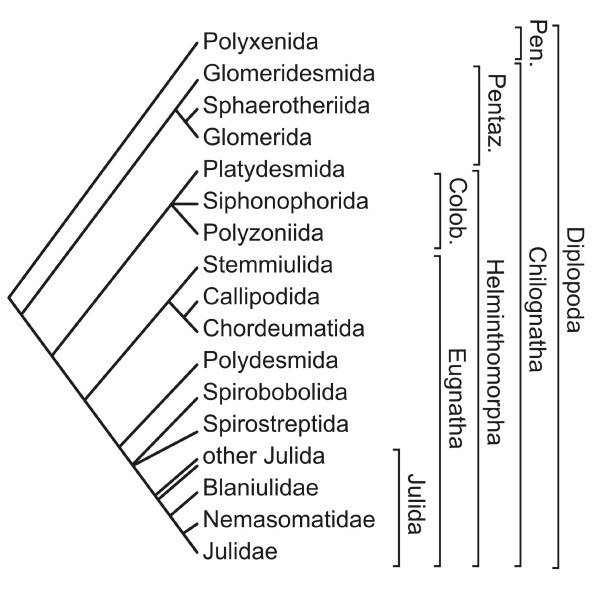
**Phylogenetic relationships among the millipede taxa cited in the text**. Colob., Colobognatha; Pen., Penicillata; Pentaz., Pentazonia. After [10,59,60].

The trunk of millipedes (Diplopoda, Figure 1) articulates into a series of segmental units to most of which (*diplosegments*) correspond two pairs of walking legs, while the first four or five post-cephalic units exhibit a different correspondence between dorsal and ventral structures, the most common arrangement being a legless *collum*, followed by three *haplosegments *with one leg pair each. Because of the independent segmentation of dorsal and ventral structures during millipede embryogenesis [1], segmental units we will refer to here are exclusively intended as descriptive modules, thus disregarding problems of homology stemming from their developmental origin [2,3].

The tiny pin-cushion millipedes, the Penicillata (= Polyxenida), have no specialized sexual appendages, while in the sister clade, the Chilognatha, specialized sexual appendages are found in adult males. The Chilognatha include two major clades, the Pentazonia, i.e. the pill millipedes (Glomerida) with their closest relatives (Glomeridesmida and Sphaerotheriida), and the Helminthomorpha, which includes the vast majority of the Diplopoda, e.g. the widespread juloid and polydesmoid forms. In the adult males of Pentazonia the last pair of appendages are replaced by a pair of articulated pincers (the *telopods*). In the adult males of the Helminthomorpha, one or two pairs of legs in the anterior part of the trunk are replaced by sexual appendages called *gonopods *[4]. These are used as claspers or to transfer sperm during copula [5].

In most Helminthomorpha, dorsal, pleural, and ventral sclerites of each segmental unit of the trunk are fused to form an exoskeletal *ring*. In the vast majority of helminthomorph millipedes the gonopods correspond to the anterior pair, or to both pairs of appendages of trunk ring VII. These differentiate in an advanced phase of post-embryonic development, first developing as normal walking legs, to change later into extremely reduced primordia, and eventually into gonopods, at subsequent post-embryonic stages. These transformations are basically limited to the appendages of the seventh trunk ring, while leaving unaffected the other rings, including those contiguous to it, in a process we have characterized as a form of *non-systemic metamorphosis *[6]. In the present study we describe some aspects of these dramatic changes affecting ring VII and its appendages in four helminthomorph species, with emphasis on the changes accompanying the development of internal structures.

In different major clades of helminthomorph millipedes, the changes in the appendages of male ring VII (leg pairs 8 and 9 in females) are quite diverse. In the Polydesmida and Callipodida, only legs 8 are replaced by gonopods, whereas legs 9 maintain their original structure and function. Similarly, in most Spirostreptida only legs 8 are replaced by gonopods, while legs 9 are completely or extensively atrophied [7]. In the remaining clades, both appendage pairs of ring VII are transformed in adult males. In many Chordeumatida, less dramatic modifications may in addition affect legs 7 and 10 (more rarely, 11 too) [8]. Finally, in Colobognatha the appendages modified into gonopods are those of pairs 9 and 10 [9].

In millipedes, the genital opening is not located on the same ring as the gonopods, but on the third trunk unit, four rings anterior to the gonopods in the Helminthomorpha. Before copulation, helminthomorph males must thus transfer sperm to gonopods, a duty generally accomplished by bending the trunk so as to bring the ventral surfaces of the third and the seventh ring closer.

Gonopod differentiation needs to be set in the context of the post-embryonic changes in trunk segmentation. The post-embryonic development of millipedes is by *anamorphosis*, i.e., the young hatch with fewer segments than the adult, and usually have three pairs of legs only. Through a series of moults, the numbers of segments and leg pairs progressively increase by the addition of new segments at the posterior end of the body. In millipedes, newly added segments generally first appear as apodous, to differentiate their legs at a following stadium [8]. Three different types of anamorphosis are distinguished in the Diplopoda [10,11]. In *euanamorphosis*, typical of the Julida and Colobognatha, moulting and the addition of new segments at each moult continue throughout the life of the animal. In *hemianamorphosis*, the post-embryonic developmental mode of Penicillata and Pentazonia, moulting continues throughout the life of the millipede, but the addition of segments stops when a final, fixed number of segments is reached. Finally, in *teloanamorphosis*, typical of Chordeumatida and Polydesmida, both moulting and segments addition stop when a fixed number of segments is reached with the moult to the final and single adult stadium.

Early descriptions of the replacement of ordinary legs by gonopods in male millipedes are, with a few others, found in Fabre [12], Brölemann [13], Verhoeff [14], Miley [15] and Davenport et al. [16]; more recent and detailed accounts have been provided by Demange [7] for some spirostreptids, Berns [17] and Dhaenens and VandenSpiegel [18] for two spirobolid millipedes, and Petit [19,20], and Filka and Shelley [21] for some polydesmidans.

Morphological changes in the external shape of gonopod primordia are often abrupt, as in the species described in the present paper, where only minor changes in size and shape of the primordia, and the surrounding area, are noticeable before the final dramatic structural leap from the most advanced anlagen to the fully differentiated gonopods. However, in other millipedes, the change from the initially tiny gonopod primordia to the definitive functional gonopods is gradually accomplished across several stadia, as in the case of the spirobolidan *Pelmatojulus insignis *(de Saussure, 1860) ([22,23] sub *Pachybolus ligulatus*). Interestingly, in some species of julidan millipedes, the differentiation of male gonopods is to some extent reversible; that is, a mature male may undergo a moult to an 'intercalary' stadium with dedifferentiated gonopods, eventually followed by another moult leading it to a second reproductive stadium with newly differentiated gonopods. This developmental mode is called *periodomorphosis *[24-28], but this morphological alternation has not been investigated in the present study.

In addition to a series of scattered accounts restricted to individual species (the polydesmid *Scytonotus virginicus *(Loomis, 1943) [29], the spirobolidan *Atopetholus angelus *Chamberlin, 1920 [30] and the spirostreptidan *Phyllogonostreptus nigrolabiatus *(Newport, 1844) [31]) the musculature of the gonopods has been described in representatives of several major groups of helminthomorph millipedes by Demange [7]. However, we know nothing of the internal changes accompanying the transformation of ordinary legs into gonopods. The only experimental studies hitherto conducted on this developmental system are the ablation/regeneration experiments carried out by Petit [19] on leg pair 8 of *Polydesmus angustus *Latzel, 1884 at stadium III. The highest degree of completeness of the regenerated appendage was obtained when the leg was cut at least 30 days before the moult to stadium IV. The regenerated appendage was gonopod-like if the level of cut was equal or distal to the apical end of the prefemur, but intermediate or definitely leg-like if more proximal.

In the present paper we describe the morphological and anatomical changes in the structures of ring VII in the males of four millipede species: two blaniulid julidans *Nopoiulus kochii *(Gervais, 1847) and *Blaniulus guttulatus *(Bosc, 1792), the nemasomatid julidan *Nemasoma varicorne *C.L. Koch, 1847, and the paradoxosomatid polydesmidan *Oxidus gracilis *(C.L. Koch, 1847). Descriptions begin with the last (in two species, the only) stadium where the appendages subsequently modified into gonopods are still represented by walking legs, to end with the first (in one species, the only) adult stadium with fully differentiated gonopods. Description of the external morphology and the internal anatomy are intended as the necessary prerequisite for more detailed investigations at the level of cell dynamics (apoptosis, proliferation) and differentiation and eventually to the level of developmental gene expression.

**Figure 2:**
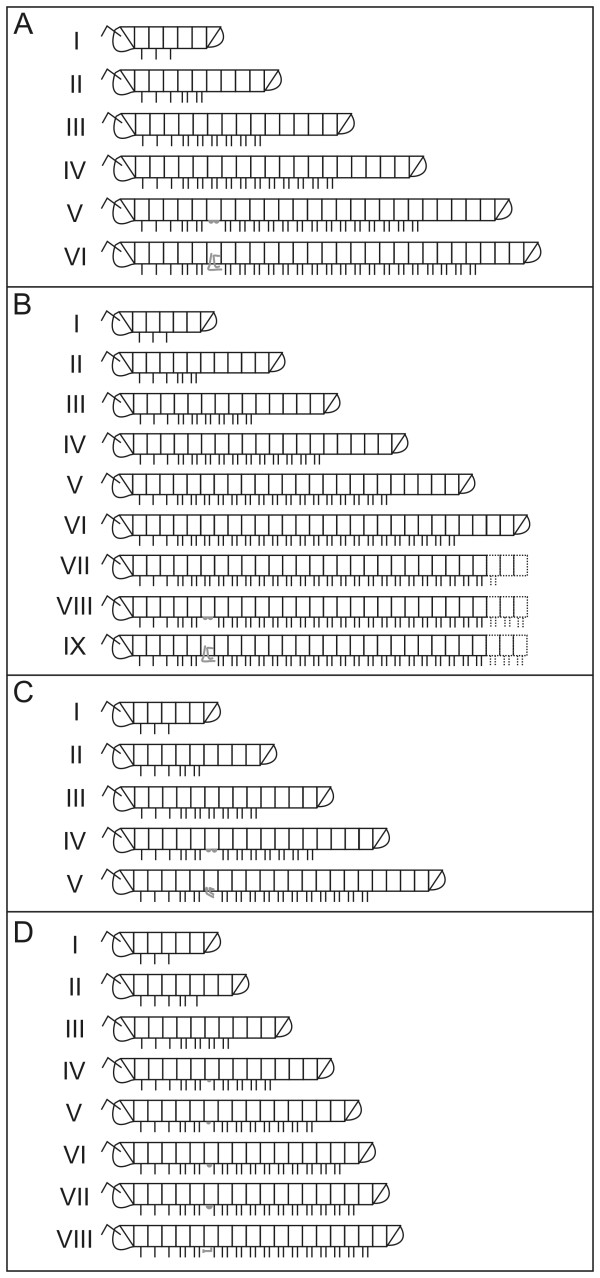
**Anamorphic development of the millipede species studied in the present paper**. The post-embryonic developmental stadia are numbered progressively. Walking legs are depicted as short lines, ventrally attached to the corresponding trunk ring, gonopod primordia as small ventral blobs on trunk ring VII, gonopods (two pairs in A-C, one pair in D) as complex structures also attached to trunk ring VII. (**A**) *Nopoiulus kochii*. (**B**) *Blaniulus guttulatus*. (**C**) *Nemasoma varicorne*. (**D**) *Oxidus gracilis*. From stadium VI on, in *B. guttulatus *only the anterior trunk is shown; in *N varicorne*, gonopods are sometimes formed at stadium VI.

The three julidan species have euanamorphic development, as is typical of the clade, and both pairs of appendages of the seventh ring of adult males are modified into gonopods (Figure 2A-C). The number of stadia prior to the achievement of sexual maturity varies with species (see Kheirallah et al. [32] for *N. kochii*, Brookes and Willoughby [33] for *B. guttulatus*, and Brölemann [34] for *N. varicorne*). For these species, a description of the external morphology of gonopod primordia and fully formed gonopods can be found in Brölemann [34] and Enghoff [35,36]. The morphological nomenclature adopted in the present paper is in agreement with modern descriptions of fully developed gonopods in Julida (e.g., [35,37]).

*Oxidus gracilis *has teloanamorphic development (Figure 2D): juveniles hatch with six trunk rings and three leg pairs, and adulthood is obtained after seven moults. Each stadium is characterized by a defined number of leg-bearing rings and terminal apodous rings [38, sub *Orthomorpha gracilis*]. In the adult stadium, the trunk is formed by nineteen rings, with 31 pairs of appendages in the female. As is typical of Polydesmida, only the first pair of appendages of the seventh ring of adult males is modified into gonopods, while the second pair does not undergo any transformation. For this species, only morphological descriptions of fully formed gonopods are available [38,39]. In the present paper, we follow the morphological nomenclature in use for taxonomic descriptions of fully developed gonopods in Polydesmida (e.g., [39-41]).

The problem of tracing homologies between the articles (podomeres) of the walking legs and the different and often inarticulated parts of the gonopods has not been resolved generally [7]. Petit [20] used setae on gonopod primordia as a set of positional markers to bridge the morphological gap between walking legs and gonopods in *Polydesmus angustus *and *Brachydesmus superus*, but this approach is not applicable to other taxa, e.g. to the julidans described in the present paper, or to the spirobolidans, as noted by Berns [17].

As documented here for the first time, the external modifications caused by the non-systemic metamorphosis of diplopods are associated with a huge rearrangement of internal anatomy.

## Results

For each stadium, specimens of different age were studied: no age-related differences were found within each stadium, apart from the last days before ecdysis, when, as the animal enters the moult period, the new cuticle becomes visible under the old one (pharate phase).

### Nopoiulus kochii

**Figure 3:**
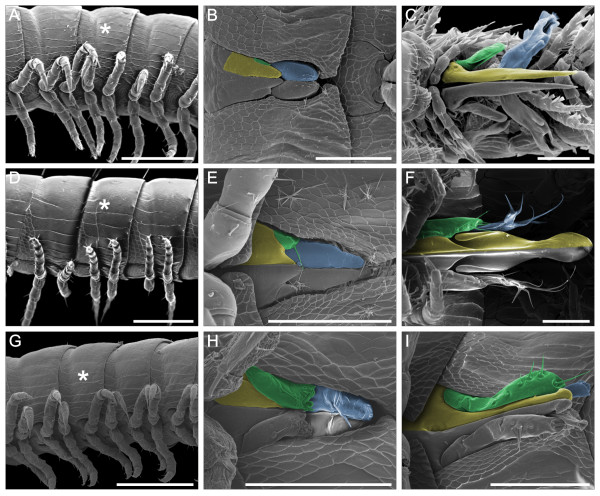
**Main stages of non-systemic metamorphosis in three millipede species of the order Julida**. (**A**-**C**) *N. kochii*; (**D**-**F**) *B. guttulatus*; (**G**-**I**) *N. varicorne*. A, D and G, last stadium with walking legs on ring VII (asterisk); SEM, latero-ventral view. B, E and H, gonopod primordia; SEM, ventral view. C, F and I, gonopods; SEM, ventral view. Yellow, coxal process; Green, anterior gonopod telopodite; Blue, posterior gonopod telopodite. Anterior to the left, scale bars 200 μm.

**Figure 4:**
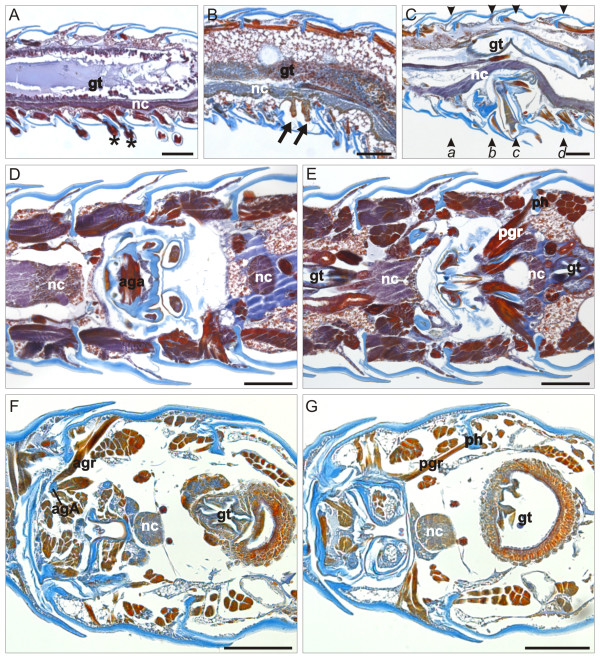
**Julida ring VII sections**. (**A**-**E**) *N. kochii *male, para-midsagittal (A-C) and frontal (D-E) paraffin sections of rings VI-VIII during post-embryonic stadia IV-VI. Mallory's triple stain. A, last stadium with walking legs on ring VII. B, stadium with gonopod primordia. C-E, adults with gonopods. (**F**-**G**) *N. varicorne *male, frontal paraffin sections of rings VI-VIII during post-embryonic stadium V. Mallory's triple stain. Labels *a-d *in panel C mark the levels of sections in Figure 9. agA, anterior gonopod apodeme; aga, anterior gonopod abductor muscle; agr, anterior gonopod retractor muscle; gt, gut; nc, nerve cord; pgr, posterior gonopod retractor muscle; ph, prophragma; arrows, possibly undifferentiated tissue. Asterisks, ring VII legs. Anterior to the left, scale bars 100 μm.

**Figure 5:**
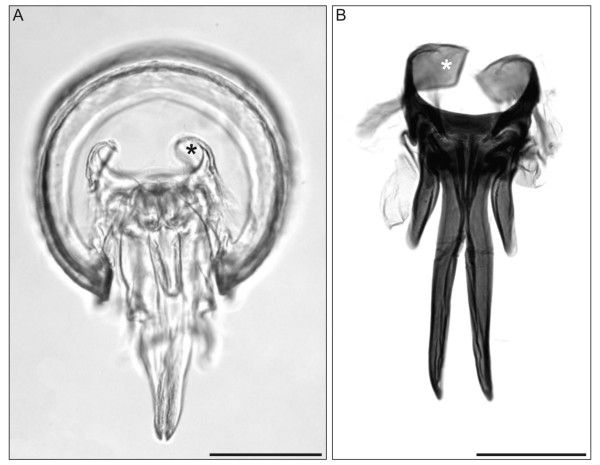
***N. kochii*, adult male**. Fused EDF-stack. (**A**) Seventh trunk ring, after digestion of internal soft tissues. Posterior view. (**B**) Dissected anterior gonopods after digestion of soft tissues. Chlorazol black stain. Asterisks, anterior gonopod apodemes. Ventral view, anterior to the top, scale bars 200 μm.

**Figure 6:**
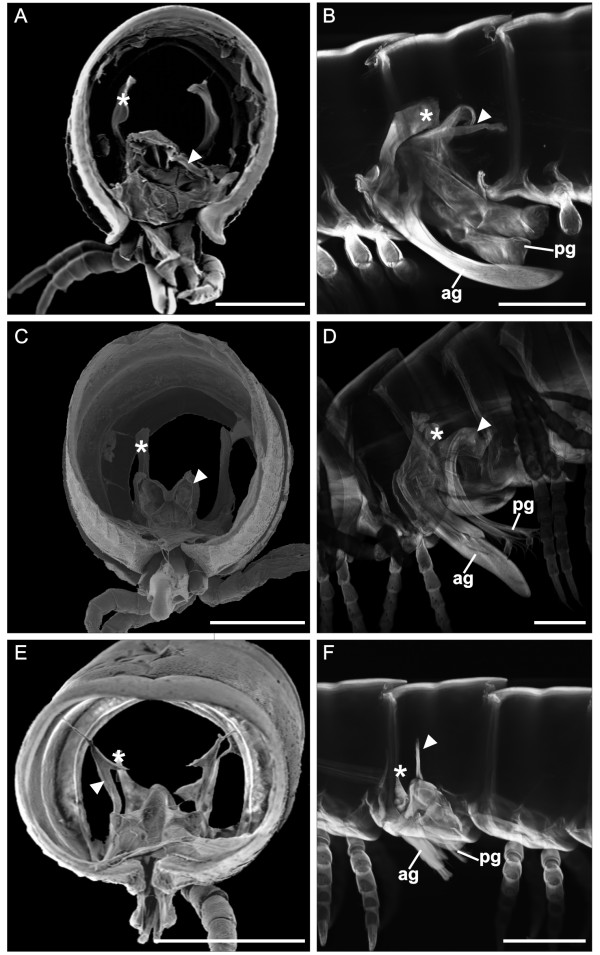
**Adult males of julidan millipedes**. (**A**-**B**) *N. kochii*, (**C**-**D**) *B. guttulatus*. (**E**-**F**) *N. varicorne*. A, C and E, seventh trunk ring, SEM after the digestion of the internal soft tissue; posterior view. B, D, and F, CLSM after digestion of the internal soft tissue. Projection at maximum intensity of serial pictures. ag, anterior gonopod; pg, posterior gonopod. Arrowhead, posterior gonopod apodeme. Asterisk, anterior gonopod apodeme. Para-midsagittal view, anterior to the left, scale bars 200 μm.

**Figure 7:**
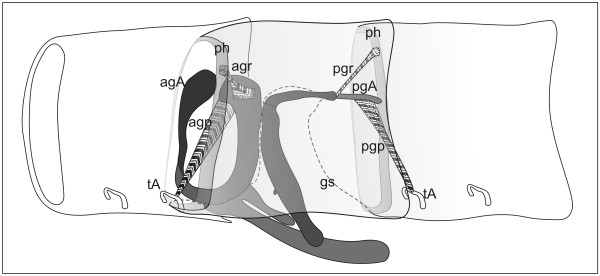
**Schematic representation of the extrinsic musculature of the left gonopods of *N. kochii***. agA, anterior gonopod apodeme; agp, anterior gonopod protractor muscle; agr, anterior gonopod retractor muscle; gs, gonopodal sac; pgA, posterior gonopod apodeme; pgp, posterior gonopod protractor muscle; pgr, posterior gonopod retractor muscle; ph, prophragma; tA, tracheal pouch apodeme. Anterior to the left.

**Figure 8:**
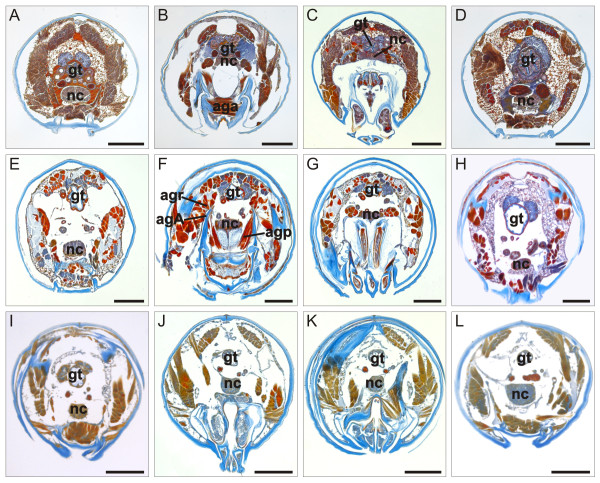
**Transverse trunk sections of adult julidan millipedes**. Mallory's triple stain. (**A**-**D**) *N. kochii*. (**E**-**H**) *B. guttulatus*. (**I**-**L**) *N. varicorne*. Sections A, E and I are at the level marked as *a *in Figure 8C; sections B, F and J are at level *b*, sections C, G and K are at level *c*, and sections D, H and L are at level *d *of the same figure. agA, anterior gonopod apodeme; aga, anterior gonopod abductor muscle; agp, anterior gonopod protractor muscle; agr, anterior gonopod retractor muscle; gt, gut; nc, nerve cord. Scale bars 100 μm.

**Figure 9:**
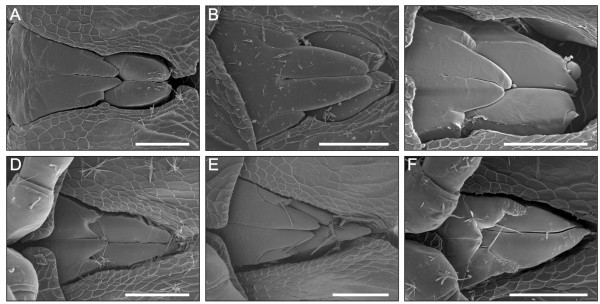
**Individual variation and left-right asymmetry in the shape of gonopod primordia**. (**A**-**C**) *N. kochii*. (**D**-**F**) *B. guttulatus*. Ventral view, anterior to the left, scale bars 50 μm.

During stadia III and IV, the two leg pairs of the seventh trunk ring are identical to the ordinary leg pairs of the other trunk rings. These legs are replaced by two pairs of gonopod primordia at stadium V, and are then changed into two pairs of bulky complex gonopods following the further moult to stadium VI (Figures 2A, 3A-C, 4A-C, 5, 6A-B, 7, 8A-D, 9A-C; cf. [6]).

Non-systemic metamorphosis is not so strictly fixed in time with respect to the animal's post-embryonic developmental schedule, as in some individuals the process actually starts one stadium later [32]. In the studied population, we recorded this exception in two cases only (< 4%, N = 53), and these males are not considered further in the present work.

In this species, males with completely formed gonopods at stadium VI are in some cases not fully reproductive [32], because the first pair of legs have not yet acquired the functional hooked shape. These stadium VI males with ordinary (non-hooked) first legs are not able to copulate, despite the presence of fully developed gonopods. In the only case of non-reproductive stadium VI males we could examine, no difference between this male and its mature companion was recorded in gonopod morphology and anatomy at the level of the seventh ring.

The gonopod primordia of *N. kochii *are two pairs of tiny laminar structures that extend in the anteroposterior direction along the ventral side of the seventh ring (Figures 3B, 9A-C). In the anterior pair of gonopod primordia the conspicuous *coxal article *elongates in the posterior direction, while the smaller *telopodite *emerges laterally. At the same time, the posterior pair of gonopod primordia, with evident telopodites, are borne on the sternite area covered by the anterior gonopod primordia. Inside each gonopod primordium, a cluster of tightly packed cells is present, possibly representing undifferentiated tissue (Figure 4B). No muscles are found in association with these appendages. There is some degree of individual variation in the detailed shape of primordia, and sizable left-right asymmetry between the two primordia was recorded in all cases examined (N = 6, Figure 9A-C).

During stadium VI, the exoskeleton area surrounding the base of the gonopods is replaced by a thin layer of cuticle which delimits the *gonopodal sac*, an exoskeletal medio-ventral invagination that shelters the basal part of gonopods (Figures 4C, 7). This dramatic change is associated with an impressive reduction of the internal ring lumen: when the gonopods are in resting position, the gonopodal sac occupies more than half of the ring volume (Figures 4C, 5A, 6A-B, 8B-C).

The anterior gonopods are composed of a long, clavate *coxal process*, accompanied laterally by a short telopodite devoid of setae (Figure 3C). The coxal process is almost totally exposed on the ventral side of the ring, and cannot be retracted into the gonopodal sac. The single U-shaped apodeme of the anterior gonopods is found at the base of the coxal processes. This huge apodeme is a prominent element in the lumen of ring VII (Figures 5, 6A-B), its anterior part also invading the posterior volume of ring VI. Two bulky muscles (anterior gonopod protractor and anterior gonopod retractor) insert on each arm of the apodeme, close to its dorsal end. The protractor muscle, which inserts on the ventral side of the anterior gonopod apodeme, is attached on the apodeme of the posterior tracheal pouch of the sixth ring. The retractor muscle, which inserts on the dorsal side of the anterior gonopod apodeme, is attached in a dorso-lateral position to the margin of the *prophragma *(anterior apodemal rim) of the seventh ring (Figure 7). A third, large, unpaired muscle (anterior gonopod abductor) connects the bases of the coxal processes of the two sides (Figures 4D, 8B) and is responsible for their abduction.

The posterior gonopods are composed of two articles, a *gonocoxa *and a *telopodite *(Figures 3C, 7). Some authors (e.g., [35]) use the word 'sternum' to indicate the gonopod article called here a gonocoxa. We prefer not to use the term sternum, to avoid confusion with the area possibly homologous to the sternite area which might be a part of the wall of the gonopodal sac. The telopodite forms proximally a knee joint with the gonocoxa and possesses two characteristic lamellae in the apical part: the *lateral lamella *and the *mesal lamella*. The lateral lamella is bordered by spines, while the mesal lamella seems to protect the other. A third and smaller *internal lamella *emerges on the mesal side of the article, mid-way of its length. Two extrinsic muscles (posterior gonopod protractor and posterior gonopod retractor) move the gonopod with respect to the trunk. The posterior gonopod retractor muscle is inserted at the base of the gonocoxa, and is attached in a dorso-lateral position to the anterior side of the prophragma (Figures 4E, 7) of the eighth ring. The posterior gonopod protractor muscle is inserted on a long gonopod apodeme that extends posteriorly from the base of the gonocoxa, and is attached on the apodeme of the anterior tracheal pouch of the eighth ring. The contraction of the protractor muscle causes the eversion of the posterior gonopod from the gonopodal sac.

The presence of voluminous anterior and posterior gonopod apodemes and of their bulky muscles causes a further reduction in the volume available for the other trunk organs. The ventral nerve cord and the digestive tract are the most influenced by these changes as they are displaced dorsally, compared with other rings in the adult and even with the same ring in immature stadia, with a consequent reduction in the gut lumen (Figures 4A-C, 8A-D).

### Blaniulus guttulatus

The development of the seventh ring in *B. guttulatus *(Figure 3D-F) is similar to the corresponding process in *N. kochii*, but shifted to later stadia: in this species, the gonopod primordia only appear at stadium VIII.

Gonopod primordia (Figures 3E, 9D-F) are similar to those of *N. kochii*, except for the presence of a seta at the apex of the primordium of the anterior gonopod telopodite. Also similarly to *N. kochii*, we found significant shape variation in gonopod primordia, both between individuals and between left and right primordia in the same animal, in particular at the level of the anterior gonopod telopodite. Histological investigation did not reveal differences in the internal anatomy associated with this external variation. The internal anatomy is very similar to that of *N. kochii*, with a cluster of tightly packed cells inside each gonopod primordium.

In adult males (stadium IX) the general architecture of the seventh ring is the same as in *N. kochii*, with a gonopodal sac that occupies more than a half of the ring volume. The two coxal processes of the anterior gonopod are fused into a single element, which continues distally into the two short telopodites, each with a few apical setae (Figure 3F).

Adult anterior gonopods are associated with a U-shaped apodeme (Figure 6C-D) which offers insertion points for a pair of antagonistic muscles (protractor and retractor) on each arm (Figure 8F). At variance with *N. kochii*, no abductor muscle is found at the base of coxal processes, because in this species, due to the fusion of the anterior gonopod coxal processes, abduction movement is no longer possible. The apodemes are less voluminous than in *N. kochii *and the two arms are less divergent.

The posterior gonopods are composed of a telopodite connected to the gonocoxa. Both articles are thinner than in *N. kochii*. The terminal part of the telopodite is characterized by the presence of two small lamellae, one with long spines at the distal end, the other with a few short spines only (Figure 3F). The trunk muscles inserting on the gonopods are organized in the same way as in *N. kochii*.

The non-systemic metamorphosis from juvenile to adult causes considerable reduction in the internal volume of the seventh ring, with respect to both the same ring in immature stadia and other adult rings. The ventral nerve cord and the digestive tract are displaced dorsally and the lumen of the gut is reduced (Figure 8E,H), but these changes are less conspicuous than in *N. kochii*.

### Nemasoma varicorne

In the males of this species, leg-pairs 8 and 9 are present at stadium III. In this stadium, trunk ring VII is identical to the other leg-bearing rings (Figure 3G). At stadium IV, these legs are replaced by gonopod primordia (see below), although in some specimens this transformation can be slightly delayed [42]. Gonopod primordia (Figure 3H) possess well-developed anterior gonopod telopodites, each with a single seta in a medial position. Posterior gonopod primordia are characterized by a transversal fold with a few short setae emerging from the proximal margin. Histological sections reveal the presence of a cluster of cells in the proximal part of each primordium. These cells seem to be less tightly packed than those observed in the corresponding appendages of the two blaniulid species.

**Figure 10:**
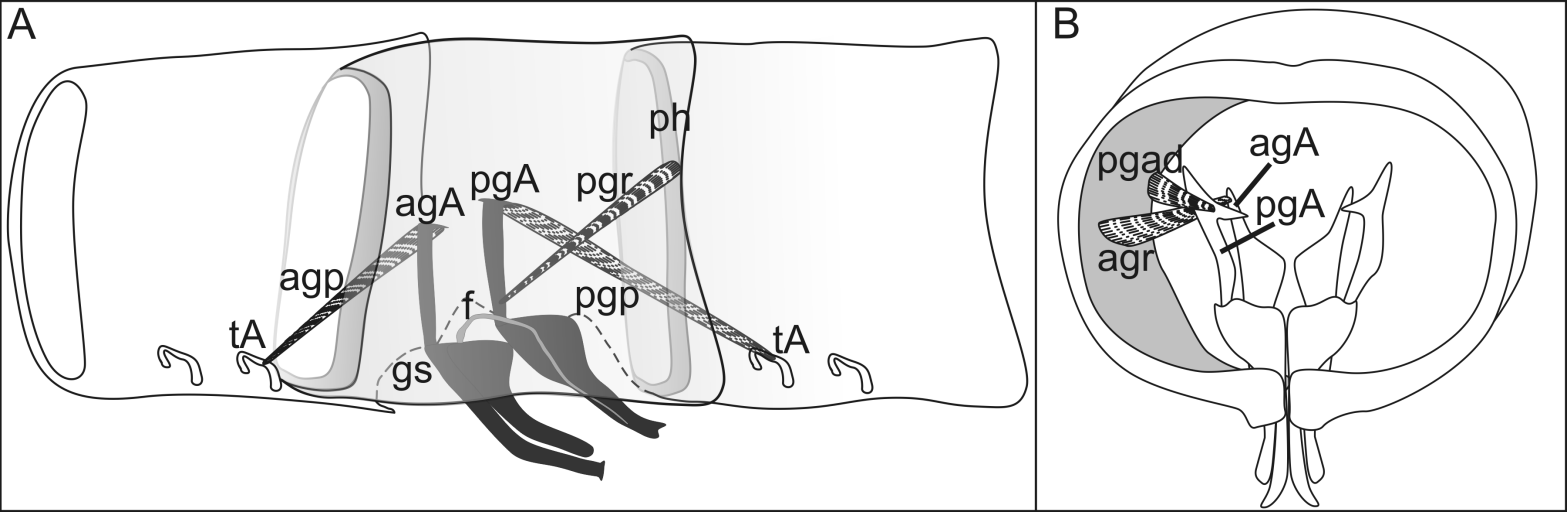
***N. varicorne *adult male**. Schematic reconstruction of the extrinsic musculature of the left anterior and posterior gonopods. (**A**) Lateral view, anterior to the left. (**B**) Posterior view. agA, anterior gonopod apodeme; agp, anterior gonopod protractor muscle; agr, anterior gonopod retractor muscle; f, flagellum; gs, gonopodal sac; pgA, posterior gonopod apodeme; pgad, posterior gonopod adductor muscle; pgp, posterior gonopod protractor muscle; pgr, posterior gonopod retractor muscle; ph, prophragma; tA, tracheal pouch apodeme.

Anterior gonopods of adult males are similar to those of the Blaniulidae (Figure 3I). The anterior gonopod is formed by one long coxal process with a hook-shaped distal end, and one long telopodite with a few setae. The telopodite is wider than the coxal process. A very elongate branch of the anterior gonopod (*flagellum*) originates from the proximo/mesal region of the anterior gonopod and extends its distal end into a groove on the distal part of the posterior gonopod of the same side (Figure 10A). A protractor muscle inserts on the extremity of a slender anterior gonopod apodeme (Figures 6E-F, 10A) and attaches to the apodeme of the posterior tracheal pouch of ring VI. The anterior gonopod retractor muscle inserts on the anterior gonopod apodeme and is attached to the lateral side of the same ring, slightly posterior to the apodeme (Figures 4F, 6E-F, 10B). A third unpaired muscle (anterior gonopod abductor) connects the bases of the coxal processes of the two sides and is responsible for their abduction.

The posterior gonopods are formed by a large basal structure that ends with a thin telopodite, almost completely hidden behind the anterior gonopods when in resting position (Figure 3I). A protractor muscle inserts on the extremity of a gonopod apodeme (Figure 10A) and attaches to the tracheal pouch apodeme of the leg pair 10 of the same side in the following ring. The gonopod retractor muscle is inserted at the base of the same posterior apodeme, and is attached to the anterior side of the prophragma of ring VIII (Figures 4G, 10A). A third muscle (gonopod adductor) inserts on the same side of the protractor muscle, to attach to the lateral side of the same ring, slightly anterior to the apodeme (Figure 10B).

The gonopodal sac is smaller than in the two Blaniulidae, and also the gonopod apodemes are less developed. As a result, the impact of gonopod development on the ring volume is less conspicuous than in the two previous species (Figure 8I-L).

### Oxidus gracilis

**Figure 11:**
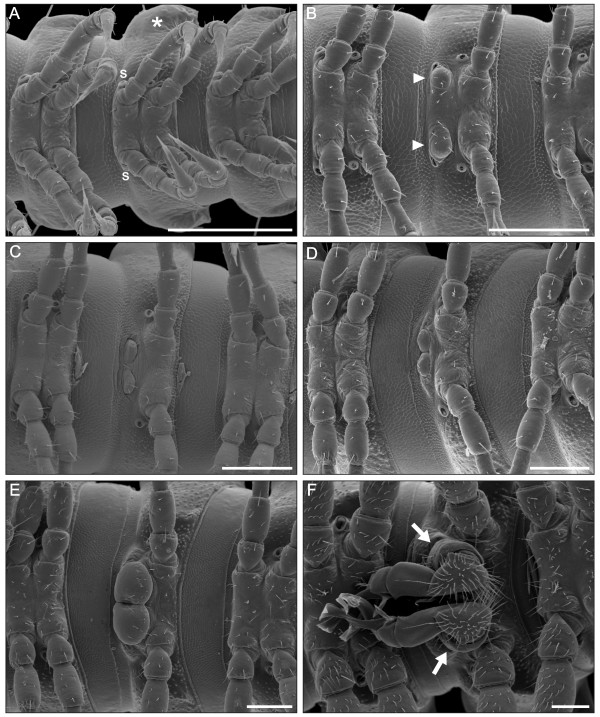
**Trunk rings VI-VIII of *O. gracilis *during developmental stadia III-VIII**. All male specimens except for the one in A, whose sex is undetermined because of the lack of sexual dimorphism at the corresponding developmental stadium. Ventral views, SEM. (**A**) Stadium III. (**B**) Stadium IV. (**C**) Stadium V. (**D**) Stadium VI. (**E**) Stadium VII. (**F**) Stadium VIII. Asterisk, trunk ring VII; s, spiracles associated with the eighth pair of appendages; arrowheads, gonopod primordia at their first appearance; arrows, gonopods. Anterior to the left, scale bars 200 μm.

At the second post-embryonic stadium, trunk ring VII is already distinct, but bears no appendages (Figure 2D). These appear with the following moult as leg pairs 8 and 9. During stadium III, the trunk is composed of eleven rings, with eleven pairs of legs in both sexes. Until the following moult, males and females are indistinguishable in external morphology. Trunk ring VII is identical to the other rings (Figure 11A), with two pairs of conventional locomotory appendages. The two pairs of spiracles open on each ring's ventral plate (or diplosternite), slightly anterior and lateral to the coxae.

From stadium IV on, it is possible to distinguish males from females because of their very different eighth appendages, which in the male are a pair of tiny rounded appendages, the gonopod primordia, which occupy the same position as the coxae of the female legs (Figure 11B). At this stadium, gonopod primordia are medially separated by a large gap. The anterior pair of spiracles in this ring are now slightly deformed, showing an oval rather than circular shape.

From stadium V to VII, the sternal surface between the gonopod primordia is progressively reduced (Figure 11C-E). After each moult, these structures appear bigger, closer, and more distinctly sunk into the sternite than in the previous stadia. The anterior pair of spiracles has disappeared since stadium V.

**Figure 12:**
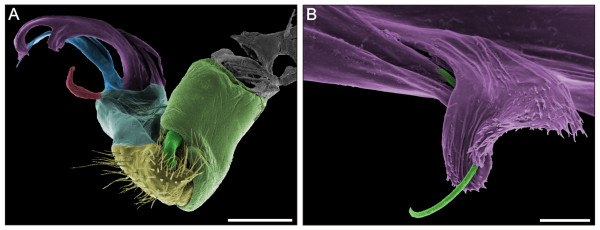
**Left male gonopod of *O. gracilis***. (**A**) Mesal view, anterior to the left. (**B**) Solenomerite and medial branch of the solenophore of the telopodite, lateral view, anterior to the right. False colour SEM. Pale green, gonocoxa; Green, solenomerite; Yellow, prefemur; Pale blue, base of the telopodite; Red, tibiotarsus; Blue, femoral process; Violet, solenophore. Scale bars: A, 200 μm; B, 20 μm.

With the seventh and last moult, males achieve the adult condition (stadium VIII). Gonopods are finally formed (Figures 11F, 12). These appendages consist of the basal gonocoxa, followed by the *prefemur *and the distal *acropodite*, prefemur and acropodite together forming the telopodite. The gonocoxa has a cylindrical shape and is basally articulated to the sternite; distally it bears a large prefemur and an elongated *solenomerite*. The solenomerite fits into a small depression at the base of the prefemur, the proximal end of the *spermatic groove*. The prefemur is entirely covered by setae. The acropodite is composed of a basal subconical part articulated to the prefemur and a complex distal part, with elongated elements: the *tibiotarsus*, the *femoral process *and the *solenophore *that surrounds the distal part of the solenomerite. The femoral process and solenophore have similar lengths; the first has a flattened end, the other ends with two branches. The medial branch is apically covered by small spines. The solenomerite is the most complex element: only the distal part is visible as a flagellar structure, partially inserted into the medial branch of the solenophore (Figure 12B). The hook-shaped tibiotarsus is situated opposite to the solenomerite.

At stadium III, no differences are found in the internal anatomy between the seventh ring and the others. The extrinsic muscles of the eighth pair of legs are in the typical arrangement described by Silvestri [43] and Manton [44] for Polydesmida. Each spiracle leads to a spacious atrium or tracheal pouch: its wall provides an apodeme for the attachment of the extrinsic muscles of the coxa.

**Figure 13:**
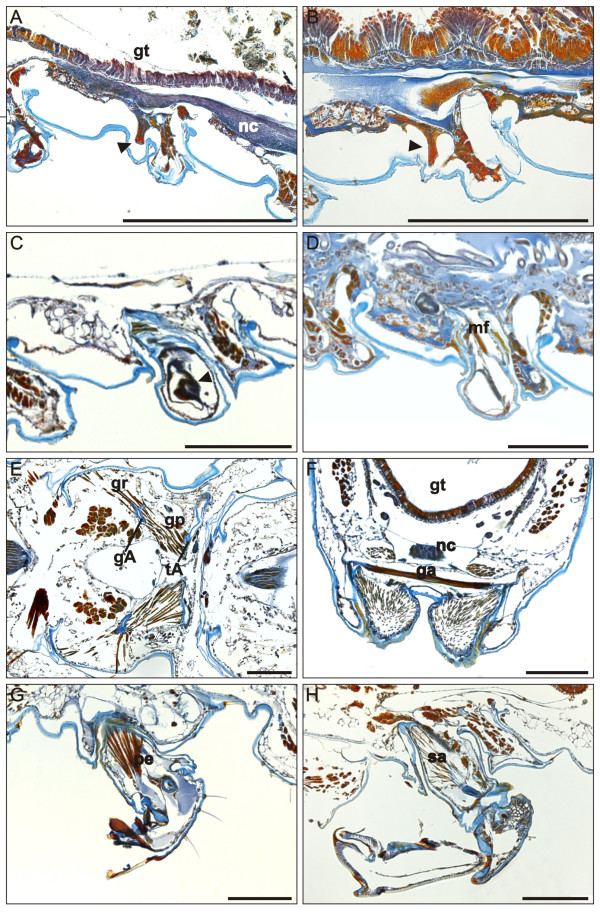
***O. gracilis *male sections**. Para-midsagittal (A-D, G-H), frontal (E) and transverse (F) paraffin sections of ring VII during stadia IV-VIII. Mallory's triple stain. (**A**) Stadium IV. (**B**) Stadium V. (**C**) Stadium VI. (**D**) Stadium VII. (**E**-**H**) Stadium VIII. gA, gonopod apodeme; ga, gonopod abductor muscle; gp, gonopod protractor muscle; gr, gonopod retractor muscle; gt, gut; mf, muscle fiber; nc, nerve cord; pe, prefemur extensor muscle; sa, solenomerite abductor muscle; tA, tracheal pouch apodeme; arrowheads, possibly undifferentiated tissue. Anterior to the left, scale bars 200 μm.

At stadium IV, the extrinsic muscles of the eighth pair of legs have disappeared, in particular the coxal (protractor and retractor) muscles that in the previous stadium attach to the tracheal pouch. The gonopod primordia contain a mass of tightly packed, not obviously differentiated cells (Figure 13A).

**Figure 14:**
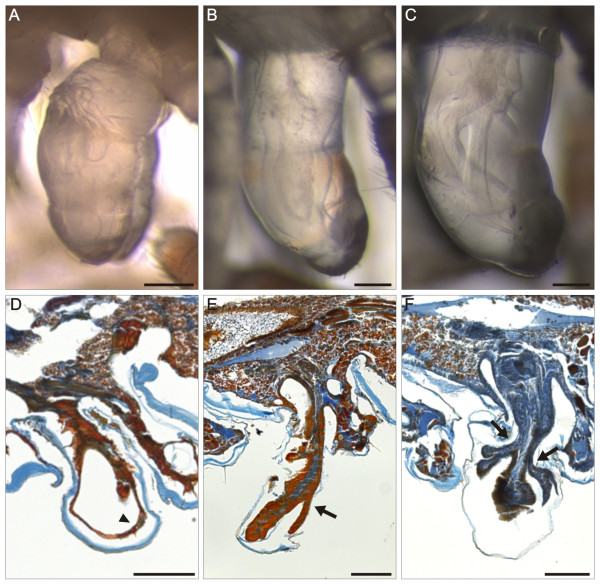
***O. gracilis *male late stadium VII (pharate phase)**. Three sub-phases are distinguishable. (**A**-**C**) Fused EDF-stacks; lateral view. (**D**-**F**) Para-midsagittal paraffin sections; Mallory's triple stain. A and D, first step of the pharate phase: the gonopod primordium is formed by three parts, separated by two constrictions; in section only the epidermis is visible (arrowhead). B and E, second step of the pharate phase: an elongated structure is visible inside the detached cuticle of the gonopod primordium; the gonopod primordium contains a mass of tightly packaged cells, distally branched (arrow). C and F, third step of the pharate phase (see text): the distal processes of the gonopod telopodite are recognizable under the transparent old cuticle; branching of distal telopodite parts is very advanced (arrows). Anterior to the left, scale bars 100 μm.

At stadium V, the internal structure of ring VII is similar to stadium IV (Figure 13B). However, in the following stadia VI and VII, tiny muscles appear proximally (Figure 13C-D), while a mass of tightly packed cells persists in the distal portion of the primordia. Behaviours associated with the moult from stadium VII to VIII (from building a molting chamber, to post-ecdysial recovery) last 12-29 days (N = 19), with an average of about 20 days, in agreement with Causey [38]. Morphologically, three steps can be distinguished on the basis of *in vivo *observations:

a) gonopod primordia are elongated in the proximodistal direction and formed of three parts, separated by two constrictions; no structure is clearly identifiable inside the primordium (Figure 14A).

b) a small increase in the length of the primordium is accompanied by the differentiation of a recognizable appendage inside the primordium (Figure 14B).

c) the distal processes of the gonopod telopodite are recognizable under the stretched cuticle of the gonopod primordia (Figure 14C).

Histological observations confirm those made *in vivo*. At the first step, only an epidermal layer is identifiable (Figure 14D). At the second step, the entire appendage contains a mass of tightly packed cells (probably undifferentiated tissue), which appears compact in the basal part, but branches distally (Figure 14E). Finally, this tissue starts to form the gonopods and in the distal part branching ends, probably corresponding to the distal parts of the telopodite, are recognizable (Figure 14F).

**Figure 15:**
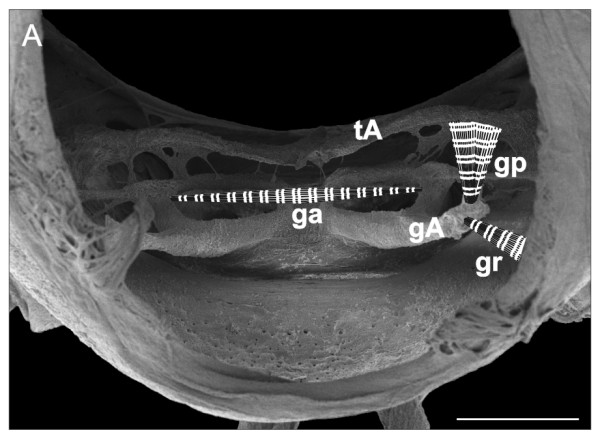
***O. gracilis *adult male**. Cuticle structure of trunk ring VII (front view) with a scheme of extrinsic musculature of left gonopod. SEM after the digestion of the internal soft tissues. gA, gonopod apodeme; ga, gonopod abductor muscle; gp, gonopod protractor muscle; gr, gonopod retractor muscle; tA, tracheal pouch apodeme. Scale bar 200 μm.

At stadium VIII (adult) the internal anatomy of the seventh trunk ring is quite different because of the changes associated with fully-developed gonopods. Each gonopod possesses an apodeme (Figure 15) that offers insertion points to a pair of antagonistic muscles, a protractor and a retractor (Figure 13E). The protractor muscles attach to the apodeme of the tracheal pouch near leg pair 9, while the retractor muscles attach on the ventro-lateral side of the same ring, slightly anterior to the apodeme. A transverse abductor muscle connects the right with the left gonocoxa (Figure 13F). The arrangement of these extrinsic muscles is summarized in Figure 15. Each gonocoxa possesses three muscles: the extensor (Figure 13G) and the flexor of the prefemur and the abductor (Figure 13H) of the solenomerite. No musculature is found inside the prefemur and acropodite (Figure 13H).

## Discussion

In many arthropods, the moult to the adult stadium is associated with major and pervasive morphological changes. These events are thus temporally restricted, but spatially unrestricted. Much less common, if not even unparalleled, is the nature of the changes which turn walking legs into gonopods in male helminthomorph millipedes. Here, indeed, the change is distributed along two to several post-embryonic stadia, but is spatially restricted to a very short, nonterminal part of the trunk. It is generally limited to one pair of appendages (leg pair 8), as in the Polydesmida, or to two pairs of appendages (usually, 8 and 9), as in the Julida, while the appendages both in front and behind those eventually turning into gonopods are totally unaffected by the event. This segmental localization of the metamorphic event is almost invariant across the Helminthomorpha, irrespective of the kind of post-embryonic development (either hemi-, telo- or euanamorphosis) and of the total number of segments in the adult millipede. Also in those few species which possess many leg pairs at hatching (e.g., more than twenty pairs in *Pachyiulus flavipes *(C.L. Koch, 1847); [10]), gonopods retain the same segmental position. This is why we described the process as a non-systemic metamorphosis [6]. The only exception to the strict localization of gonopods at trunk ring VII is the Colobognatha, where the legs modified into sexual appendages are those of pairs 9 and 10 which belong to segments VII and VIII respectively [9]. This supports the view of an independent genetic control of development between dorsal and ventral segmental units. Thus non-systemic metamorphosis should not be understood as the transformation of a 'segmental module'.

On the other hand, the reduction of the relevant ordinary legs to gonopod primordia and the change of the latter into gonopods have no universally fixed position in the millipede's post-embryonic developmental schedule. The number of stadia with gonopod primordia varies among helminthomorph millipedes, sometimes also between quite closely related taxa. For example, among the polydesmids, Petit [20] contrasted *Polydesmus angustus*, with four stadia (IV to VII) with gonopod primordia, with *Brachydesmus superus *Latzel, 1884, where these stadia are reduced to three (IV to VI). However, even more deviant schedules are possible. In *Pelmatojulus insignis*, gonopod primordia differentiate progressively from stadium IV to IX, eventually approaching the final condition in the subadult stadium X, followed at last by the functional adult as stadium XI [23].

The precise spatial localization of gonopods has suggested the involvement of a positional marker set at a quite early embryonic stage, when the general organization of the body is the same in all millipedes [6]. This hypothesis implies the presence of a 'dormant' developmental module which is activated months, or years, after it has been set up during embryogenesis. The same module would be used repeatedly in the species with periodomorphosis. The nature of the organs involved in the metamorphosis and the posterior position of the seventh diplosegment in the embryo has suggested the *Hox *gene *Abd-B *as a candidate marker [6]. Unfortunately the expression pattern of *Abd-B *has not been studied in the only helminthomorph millipede, *Oxidus gracilis*, for which data on *Hox *gene expression are available [45]. To date, millipedes have proved to be difficult subjects for the study of embryonic and, still more, post-embryonic development: post-embryonic stages are difficult to handle experimentally, because of the heavily calcified exoskeleton and also because of the disturbance to histochemical procedures caused by the diversity of repugnatory substances they produce. A comprehensive study of embryonic gene expression has been only conducted on the pill millipede *Glomeris *[1,46,47], which lacks gonopods.

Functional aspects of leg-to-gonopod transformation have not been experimentally investigated, but there are hints suggesting possible connections with the feeding capacity of sexually mature males. Specifically, sub-optimal energy intake during the stadium/stadia with gonopods (and during the pharate phase of the preceding stadium) offers a possible functional explanation for the existence of intercalary males in the species with periodomorphosis. The same hindrance to feeding, caused by displacement and reduction in the lumen of the gut in many adult julidan males, has also a possible connection with the little known observation that acquisition of sexual maturity appears to be accompanied by a 'slimming' of the body of those males, with respect to trunk diameter in pre-adult stadia [48,49].

## Conclusions

The evidence provided in this paper shows that the non-systemic metamorphosis [6] that gives rise to millipede male gonopods, while dramatically modifying the external shape of the eighth and ninth pairs of appendages, is accompanied, in some taxa, by very extensive changes in the internal anatomy of trunk ring VII. Both blaniulid species examined here show a major remodelling of the architecture of this ring, as a consequence of the reduction of the volume available to structures other than gonopods. This effect is probably amplified in these species by their overall reduced size and very small body diameter. Indeed, the remodeling of ring VII architecture is less conspicuous in the other two species, both of them of larger size. Comparative evidence from a larger sample of millipede species is obviously needed.

Our descriptions of the external and internal changes eventually replacing ordinary locomotory legs with specialized sexual appendages can be a starting point for the study of the cellular dynamics - apoptosis and proliferation processes - involved in gonopod formation. Moreover, the morpho-anatomical information that emerges from these data provide an important basis for the study of post-embryonic expression patterns of genes putatively involved this kind of metamorphosis, as suggested by Drago et al. [6].

## Materials and methods

### Animal collection

Cultures of two millipede species, known to be easily kept in captivity, were established in our lab. Adult specimens of *Nopoiulus kochii *were collected in litter and in decaying wood in Sorio di Gambellara (NE Italy), and adult specimens of *Oxidus gracilis *were collected in litter in Marcon di Venezia (NE Italy). Breeding cultures were kept at 20°C in plastic Petri dishes, with ca 5 mm of 2% agar on the bottom [42,50]. Original litter material, pieces of potatoes and decaying wood (*Populus, Salix*) were used as food. Males were reared until the selected developmental stage, when they were euthanized using ethyl acetate vapour at 4°C, to prevent the body from coiling, and immediately fixed in a mixture of 2.5% glutaraldehyde and 2% formaldehyde in PBS. For optimal fixation, the millipedes were cut in two or more pieces before immersion into the fixative, where they remained for at least 48 h at 4°C. In some cases, stereomicroscope observations on living specimens were carried out before euthanization.

Individuals of different stadia of the other two species, *Blaniulus guttulatus *and *Nemasoma varicorne*, were collected directly in the field, immediately assigned to their developmental stadium by counting the number of leg-bearing and apodous rings, then euthanized and fixed as described above. Specimens of *B. guttulatus *were collected in a garden in Hellerup (Copenhagen, Denmark), those of *N. varicorne *were collected under tree bark in Gentofte, Ermelunden (Copenhagen, Denmark) and in Jægersborg Hegn (Copenhagen, Denmark).

In addition, several specimens of the three julidan species from the collections of the Zoological Museum in Copenhagen, preserved in 70% ethanol, were studied to increase sample size in SEM investigations.

### Digestion and dissection

To examine the exoskeleton and its internal projections (apodemes), internal soft tissues of selected specimens were digested: fixed animals were washed from the fixative in PBS+0.1% Triton^®^X 100, cut in pieces and immersed in 5% KOH at 50°C for at least 48 h. The cuticle thus obtained was rinsed with distilled water and dehydrated, for SEM observations, or bleached and stained, for CLSM observations (see below). When required, gonopods of fixed or digested specimens were dissected using micro-scissors, micro-pins, scalpel and tweezers.

### Morphology

Living animals were observed using a Leica MZ125 stereomicroscope fitted with a Leica DFC 420 camera.

Digested preparations destined to bright field light microscopy were stained with Chlorazol black and examined with a Leica DM5000B microscope. Images were acquired with a Leica DFC 300 FX camera using the software Leica Application Suite (ver. 2.6) and focus-stacked using the CombineZP Image Stacking Software (developed by Alan Hadley).

Fixed specimens destined for SEM were washed from the fixative in PBS+0.1% Triton^®^X 100 then dehydrated through a graded ethanol series. For a complete dehydration the samples were immersed in hexamethyldisilazane [51], air dried at room temperature, and finally mounted on aluminium stubs. When necessary, to remove soil particles, samples were briefly sonicated, before being finally coated with gold or platinum-palladium. Three scanning electron microscopes were used: a Cambridge Stereoscan 260, a Jeol JSM-6490, and a Jeol JSM-6335-F.

In order to reconstruct the morphology of the less accessible parts of the gonopods and their associated structures (apodemes), a modern technique based on confocal laser scanning microscopy (CLSM) was adopted. This technique (see [52-55]) takes advantage of the autofluorescence of the cuticle, and allows detection without dissections of structures that are extremely difficult to handle in specimens of small size, for which dissection can easily cause damage to the parts of interest. Specimens destined for CLSM were washed in PBS+0.1% Triton^®^X 100 to remove the fixative. Internal soft tissues were digested with 5% KOH at 50°C for at least 48 h. The remaining cuticle was bleached with acetic acid and washed with distilled water. Evans Blue staining (0.005% in water) was used to increase the fluorescence of the cuticle, improving resolution beyond the effect of autofluorescence. Specimens were washed several times to remove excess stain. Samples were mounted in PBS-buffered glycerol with anti-fade agent (90% glycerol, 0.5% n-propyl gallate in PBS). Because increasing specimen thickness exacerbates aberrations in the optical setup [56,57], care was taken to use just the amount of medium necessary to cover the specimens. Slides were studied using a Nikon Eclipse E600 microscope equipped with a Bio-Rad MRC 1024ES confocal laser scanning unit using a 543 nm helium/neon laser and a 570 nm long pass emission filter. CLSM data were analyzed with the software ImageJ (ver. 1.37).

### Anatomy

Soft parts anatomy was studied through serial paraffin sections. Fixed specimens were washed in PBS+0.1% Triton^®^X 100 at least six times, each time for 20 min, and treated overnight with chitinase from *Streptomyces griseus *(0.7 u/ml at 37°C in HEPES buffer) to soften the exoskeleton; samples were then washed in PBS+0.1% Triton^®^X 100 and decalcified overnight in EDTA 0.5 M pH 8 at 4°C. After washing in PBS+0.1% Triton^®^X 100, the specimens were dehydrated through a graded ethanol series, then immersed in xylene and finally embedded in paraffin. Sagittal, frontal and transversal sections were made at a thickness from 7 to 12 μm (always 7 μm in the case of julidans) and stained with Mallory's triple stain [58]. Slides were observed with a Leica DM5000B microscope, fitted with a Leica DFC 300 FX camera.

### Image processing

Digital images were processed with the software CorelPHOTO-PAINT, CorelDRAW (ver. 14) and Adobe Photoshop (ver. 12).

## Competing interests

The authors declare that they have no competing interests.

## Authors' contributions

LD carried out the morphological investigations on non-systemic metamorphosis in the four millipede species, assisted by EG. LD, GF and AM implemented data analysis and interpretation, and drafted the article. All authors participated in the design of the study, and read and approved the final manuscript.
